# Biochemical and Histological Characterization of Sorcin Overexpression in Patients Who Underwent Radical Prostatectomy

**DOI:** 10.5152/tud.2025.24188

**Published:** 2025-06-24

**Authors:** Kenan Toprak, Mehmet Gokhan Culha, Huseyin Ozgur Kazan, Ayberk Iplikci, Gozde Kir, Gozde Ecem Cecikoglu, Ahmad Kado, Hayriye Erman, Asif Yildirim

**Affiliations:** 1 Department of Urology, Istanbul Medeniyet University Faculty of Medicine, Istanbul, Türkiye; 2 Department of Urology, Prof. Dr. Cemil Tascioglu City Hospital, University of Health Sciences, Istanbul, Türkiye; 3 Department of Pathology, Istanbul Medeniyet University Faculty of Medicine, Istanbul, Türkiye; 4 Department of Biochemistry, Istanbul Medeniyet University Faculty of Medicine, Istanbul, Türkiye

**Keywords:** Diagnosis, prognosis, prostate cancer, sorcin, staging

## Abstract

**Objective:**

Sorcin, a signaling molecule, has recently emerged as a significant focus within cancer research. This study aimed to compare histopathology results with serum sorcin level and tissue sorcin immunohistochemical expression in patients who underwent radical prostatectomy (RP).

**Methods:**

A total of 81 patients who underwent RP between December 2017 and June 2019 due to prostate cancer (PCa) and had not received any previous treatment were included in the study. Patients attended regular follow-up appointments for at least 24 months. In order to compare serum sorcin levels, the control group consisted of 67 healthy men. Demographic data of participants were recorded. In the PCa group, pathology data from both Transrectal ultrasound-guided biopsy and RP were documented.

**Results:**

Prostate-specific antigen (PSA) and sorcin levels of PCa patients were found to be higher than the control group (*P* < .001, *P* = .02). In the comparison of sorcin levels and sorcin staining percentages of PCa patients with histopathological and clinical findings; while sorcin levels were found to be higher in patients with positive lymph nodes (*P* = .018) and with biochemical recurrence (*P* = .049), no significant difference was found in any histopathological finding in terms of sorcin staining percentages. In an receiver operating characteristic curve analysis calculation for sorcin levels in PCa patients, AUC = 0.563 and when the cut-off value was taken as 0.415 ng/mL, 59.3% sensitivity 60.1% specificity was determined.

**Conclusion:**

Sorcin was found to be higher in PCa compared to healthy males. In addition, high International Society of Urological Pathology grade groups were observed in patients with high sorcin levels.

**Main Points**

- Sorcin, which has been proven to be overexpressed in many different types of cancer, was found to be higher in prostate cancer compared to healthy men in this study.
- High sorcin levels were correlated with high International Society of Urological Pathology grades.
- Sorcin may be used in the diagnosis, treatment, and follow-up of prostate cancer patients in the near future.

## Introduction

Prostate cancer (PCa) is the second most commonly diagnosed cancer in the male population. Epidemiological data suggest that there were around 1.4 million incident cases and 375 000 deaths attributable to this malignancy on a global scale.^1^ A systematic review of autopsy studies revealed a 5% prevalence of PCa in males under the age of thirty. The risk increases 1.7 times per every decade and the prevalence rises to 59% (48%-71%) in the population over 79 years of age.^2^ Family history and race have been linked to a higher incidence of PCa, indicating a potential genetic predisposition. Hereditary PCa is associated with earlier onset, but the course of the disease is similar.^3^ Many factors cause PCa to occur, including genetic and epigenetic changes. Studies in the literature have identified a vast array of signaling molecules associated with cancer, with their dysregulation playing a critical role in the initiation and progression of the disease. These signaling molecules include Akt, Erk, IκB kinase, NF-κB, STAT3, Wnt, TNFα and TNFα-induced proteins, etc.^4^^,^^5^ Some molecules have been shown to have diagnostic and therapeutic value, as many drugs targeting these key molecules have been discovered to have high therapeutic potential against different types of cancer.^6^

Sorcin (soluble resistance related calcium binding protein) is one of the signaling molecule that has recently received more attention in cancer research as it has been found to induce multidrug resistance (MDR) in cancers.^7^^,^^8^ It is a 22 kDa soluble, small, penta EF family of calcium (Ca2+)-binding protein associated with calcium (Ca2+) homeostasis, MDR, and cancer development.^8^ Sorcin was first identified by Meyers and Biedler in the vincristine resistant Chinese hamster lung cell line DC-3F/VCRd-5 L and was shown to increase drug excretion in MDR cells in a Ca2+ dependent path.^7^ Sorcin has been shown to be expressed at high levels in many human tissues, heart, brain, kidneys, breast, and skin. Sorcin has been found to be overexpressed in many cancers such as leukemia, stomach, lung, ovarian, and breast cancers.^9^ It has been shown that proapoptotic mechanisms and mitochondrial apoptosis decrease with the increase in sorcin levels. While it has been shown that sorcin levels are increased in many epithelial tumors, the relationship between PCa and sorcin has not yet been questioned in literature reviews. It was hypothesized that sorcin might be overexpressed in prostate cancer patients. Questioning the presence of sorcin in PCa, which has previously been shown to be overexpressed in colorectal carcinoma, breast, ovary, and many epithelial tumors, will help with the relationship between sorcin levels and the disease in terms of predicting the decision for follow-up or treatment.

This study aims to compare the histopathology results of serum sorcin level and tissue sorcin immunohistochemical expression in PCa patients who underwent radical prostatectomy (RP), with the goal of elucidating the correlation between sorcin and prostate cancer.

## Material and Methods

**Figure 1.**
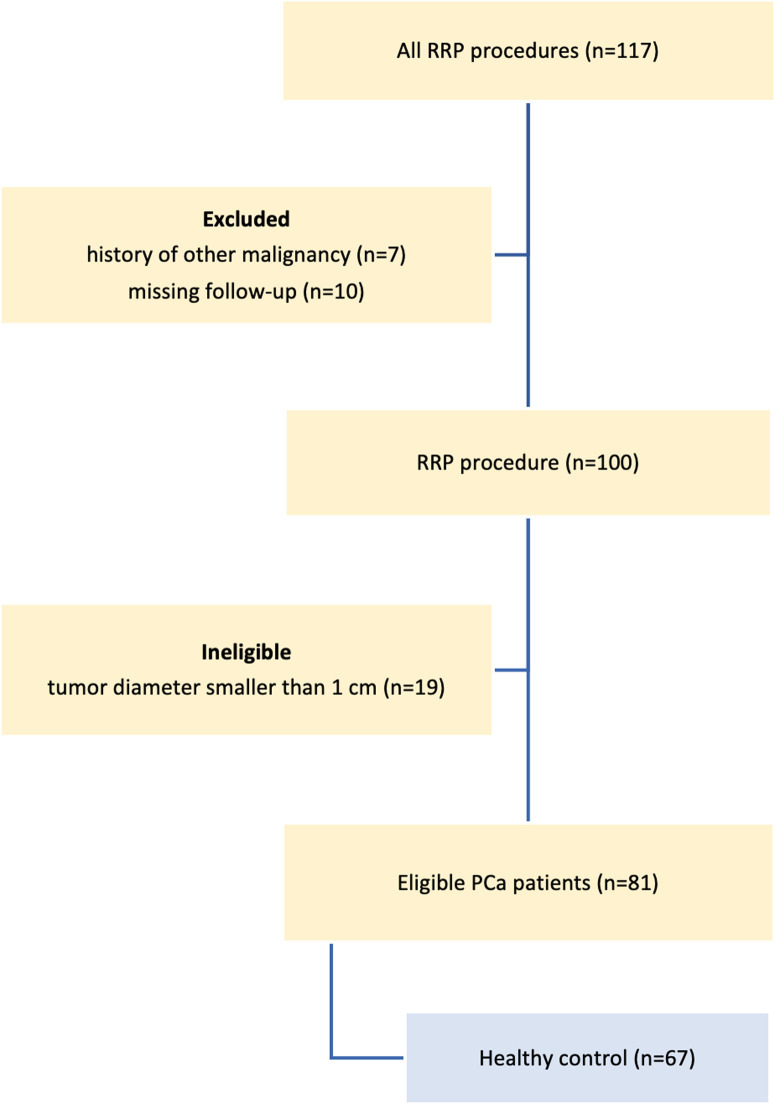
Study flowchart.

A total of 81 patients who underwent RP between December 2017 and June 2019 due to PCa and had not received any previous treatment were included in the study. All patients underwent RP as a definitive treatment and attended regular follow-up appointments for a minimum of 24 months post-procedure. All patients included in the study had suitable paraffin blocks (tumor diameter greater than 1 cm) for immunohistochemical examination. In order to compare serum sorcin levels, 67 healthy men, carefully selected to have no suspicion of PCa and no co-existing medical conditions, were included in the study. Figure 1 shows the entire cohort represented by a flow diagram. Blood samples were taken from all participants in the morning after a 6-hour fast and were stored at −80°C until the day of examination. In the PCa group, blood samples were taken before the RP procedure.

The present study protocol was reviewed and approved by the Istanbul Medeniyet University Ethical Committee (approval number: 2019/0035, date: 06/02/2019). Written informed consent was obtained from all subjects when they were enrolled.

Demographic data of the participants (age, body mass index (BMI), prostate-specific antigen (PSA)) were recorded. In the PCa group, data from both Transrectal ultrasound-guided (TRUSG) biopsy and RP pathology (Gleason score, International Society of Urological Pathology (ISUP) grade, TNM stage, presence of extraprostatic extension (EPE), perineural invasion (PNI), and lymphovascular invasion (LVI)) were documented. Sorcin level and staining were performed using the following steps.

### Measurement of Sorcin Levels

In the method based on the sandwich enzyme-linked immunosorbent assay (ELISA) principle, sorcin, the amount of which was unknown in the sample, was bound to the sorcin antibody-coated wells. The immune complexes formed by sorcin proteins with a biotinylated secondary antibody added to the medium generated a color directly proportional to the amount of sorcin in the sample as a result of the reaction with the addition of streptavidin peroxidase and tetramethylbenzidine substrate. The absorbance of the resulting color was measured at 450 nm. An ELISA commercial kit (Catalog code: E2542Hu, Human Sorcin ELISA Kit, BT LAB, Shanghai, China) was used to determine serum sorcin levels.

### Immunohistochemical Evaluation

Anti-SRI antibody was applied to 81 cases included in the study, and the protocol applied is as follows. After preparing 4 sections of 3 µm thickness, tissue sections were placed on electrostatically charged slides and dried at 60°C for at least 2 hours. The entire immunohistochemical staining process, including deparaffinization and antigen exposure, was performed on a Bond-Max Leica fully automated stainer. The primary antibody was automatically dispensed and incubated at 37°C for 30 minutes. Anti-SRI (Clone: Polyclonal, Product number: HPA073666, Atlas antibodies, Dilution: 1/100) was used as the primary antibody. Immunohistochemical staining was performed with a biotin-free, HRP multimer-based, hydrogen peroxide substrate 3’-diaminobenzidine tetrahydrochloride containing chromogen (Bond™ 30 Leica Bond Refine Detection Kit, Leica/novocastra, Buffalo Grove, IL, United States) kit with a fully automatic immunohistochemistry staining device (Bond-Max, Leica). In the device, the dehydration of the sections, whose counterstaining was completed with hematoxylin and bluing solution, along with clearing with xylene and covering with a coverslip, was performed automatically (Leica, CV 5030), and the process was terminated.

Patients were evaluated immunohistochemically for sorcin expression. Evaluations were made semi-quantitatively. For sorcin expression, the cytoplasmic and membranous staining intensity was manually scored from 0 to 3; 0 = unstained, 1 = weak staining, 2 = moderate staining, 3 = strong staining; The extent of staining was scored as 0%-100%.

### Statistical Analysis

SPSS 25.0 (IBM SPSS Corp.; Armonk, NY, USA) program was used for statistical analysis. The distribution of cases was evaluated with the Kolmogorov–Smirnov test. Descriptive statistical methods (mean, SD, median, frequency, ratio, minimum, maximum) were used while evaluating the study data. Independent sample *t*-test, Mann–Whitney *U* test, and chi-squared test were used to compare the paired groups. ANOVA test was used for multi-group evaluation. Pearson correlation test was used for correlation. Cut-off values were determined by receiver operating characteristic (ROC) curve analysis. A statistically significant *P*-value was determined as <.05.

## Results

A total of 148 participants (81 PCa and 67 controls) were enrolled in the study. The mean age of the men participating in the study was 66.07 ± 6.68 years (49-86), and the mean BMI was 26.96 ± 3.30 kg/m^2^ (19.62-34.32). The mean age of PCa patients was found to be lower than that of the control group (*P* < .001). Prostate-specific antigen levels and sorcin levels of PCa patients were found to be higher than the control group (*P* < .001 for PSA and *P* = .02 for sorcin) (Table 1).

The median follow-up for patients in the PCa group was 34 (24-42) months, and the definition of biochemical recurrence (BCR) was a rising PSA of ≥ 0.2 ng/mL after RP. When the data of 81 patients diagnosed with PCa were analyzed, pathological findings were identified in the digital rectal examination in 58% (47/81) of the patients. Surgical margin positivity was detected in a total of 25 patients (30.9%). Lymph node dissection was performed in 27 of the patients who underwent RP and positivity was detected in 4 (14.81%) of these patients. A total of 42 patients had EPE, 61 patients had PNI, and 7 patients had LVI. A cribriform pattern was found in the pathology of 50 patients and intraductal carcinoma was found in 8 patients. During the follow-up period, BCR was detected in a total of 21 patients, and one of these patients died due to metastatic disease.

**Figure 2.**
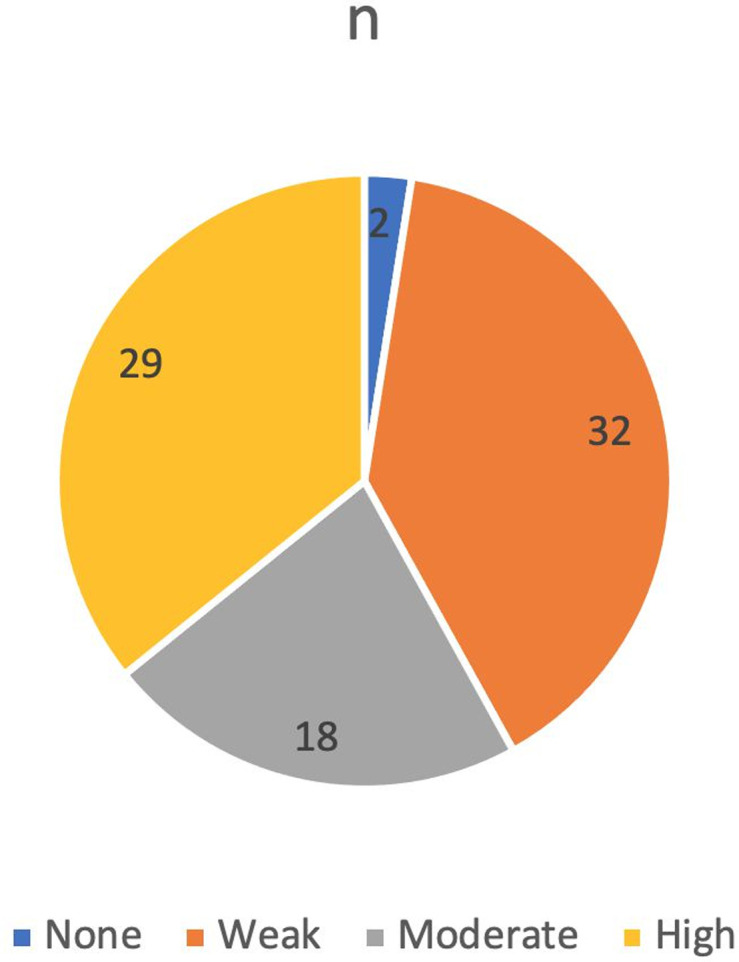
Sorcin staining strength in prostate cancer patients.

In the evaluation of the sorcin staining strength from pathology specimens of PCa patients, 35.8% strong staining, 22.2% moderate staining and 39.5% weak staining with sorcin were found. Sorcin staining was not detected in 2 cases (2.5%) (Figure 2). In the comparison of sorcin levels and sorcin staining percentages of PCa patients with histopathological and clinical findings; while serum sorcin level was found to be higher in patients with positive lymph node, patients with cribriform pattern and patients with BCR, no significant difference was found in terms of sorcin staining percentages in any histopathological finding (*P* = .018 for lymph node positivity, *P* = .02 for cribriform pattern, *P* = .049 for BCR) (Table 2).

When comparing serum sorcin levels and sorcin staining percentages with ISUP grade determined after TRUSG biopsy and RP, a significant difference was found between serum sorcin levels in TRUSG biopsy ISUP grade group, and this difference was noted for ISUP grade 5 in post-hoc analysis (Table 3).

**Figure 3.**
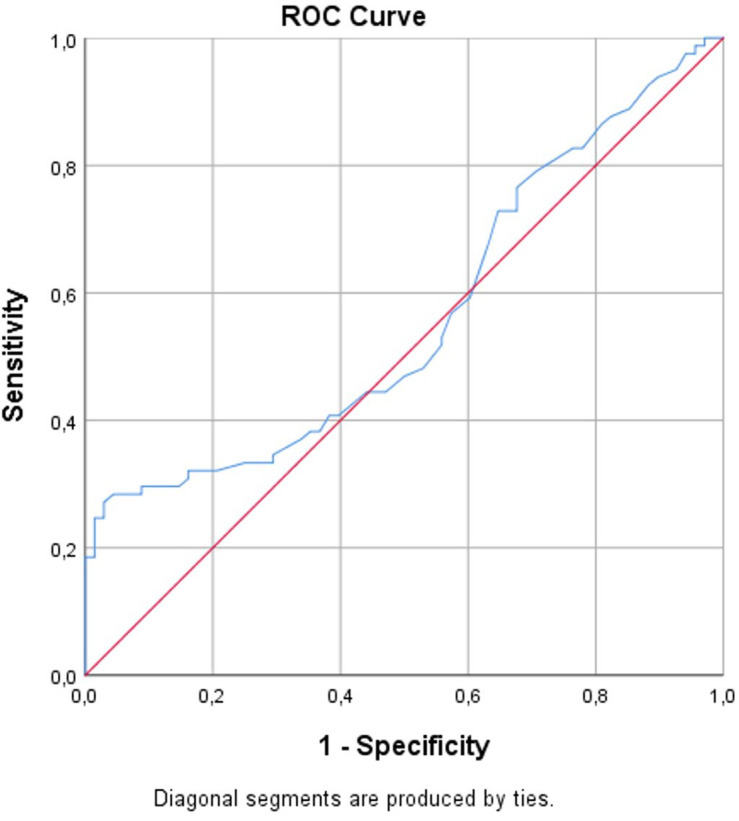
Receiver operating characteristic curve analysis for serum sorcin level to be used in the diagnosis of prostate cancer.

In the ROC curve analysis calculation for serum sorcin levels in PCa patients, the area under the curve (AUC) = 0.563 (CI: 0.471-0.656) was found, and when the cut-off value was selected as 0.415 ng/mL, 59.3% sensitivity 60.1% specificity, 58.7% positive predictive value, and 56.7% negative predictive value were found (Figure 3).

## Discussion

As a result of the study, it was determined that the serum sorcin levels of PCa patients were statistically significantly higher than those of normal healthy men, and this elevation was associated with TRUSG biopsy ISUP grade. A serum sorcin level higher than 0.415 is significant in terms of PCa risk.

Many biomolecules related to PCa risk determination have been evaluated, and studies have been conducted that reveal significant findings in terms of diagnosis, treatment, and prognosis.^6^ In this study, the higher serum sorcin level in PCa patients compared to normal men shows that serum sorcin may be used for the diagnosis of PCa.

Overexpression of sorcin has been reported in a number of tumor resistant cell lines. Growing evidence suggests that sorcin plays a role in survival mechanisms contributing to MDR and is linked to poor prognosis in cancer patients undergoing therapeutic treatment.^10^^-^^12^ There are also studies about sorcin, which has been shown in many tissues and can also be evaluated as a predictable factor for evaluating response to chemotherapy.^13^

Most studies investigating sorcin expression have used commercially available cell lines engineered using complementary DNA cloning or small interfering RNA.^14^ In addition, by examining the resistance of certain cancer cells to apoptosis, the mechanism of sorcin in the prognosis of cancer can be elucidated.^15^ In this study, it was found that the serum sorcin levels of patients with a TRUSG biopsy ISUP stage 5 in TRUSG biopsy were higher than the other stages. Overexpression of sorcin in the advanced prognosis of cancer may be a marker of poor prognosis in PCa.

Breast cancer is a leading cause of cancer-related mortality among women, and sorcin has been linked to increased morbidity and mortality in these patients.^16^ Predominantly, breast cancer cell resistance to antiblastic cells is most likely a factor causing therapeutic failure. Since sorcin is recognized as an important protein related to breast cancer resistance, understanding the mechanisms of sorcin at the molecular level may have a significant impact on the clinical management of patients with PCa, which has many aspects similar to breast cancer.

The exact role of sorcin remains incompletely understood. However, sorcin has been observed to help regulate homeostasis, apoptosis, and vesicle transfer in cells.^17^ Sorcin has an important role in the regulation of calcium (Ca2+) homeostasis in the human body. Calcium (Ca2+) plays important roles in neurons, including synaptic plasticity and apoptosis, and the release of this neuronal calcium signaling is known to be one of the central mechanisms of different neurodegenerative diseases such as Alzheimer’s disease.^18^

Invasion and migration are the 2 main manifestations of tumor progression. Many studies have delineated 2 models of invasion in cancer: individual cell migration and collective cell migration, wherein tumor cells can traverse the extracellular matrix barrier and disseminate to adjacent tissues. In addition, cancer is associated with several critical processes, including the inhibition of apoptosis, MDR, and epithelial-mesenchymal transition. It can also regulate various oncogenic genes involved in these processes, such as sorcin and phosphorylated ERK.^19^ In this study, it was determined that sorcin levels were higher in PCa patients with lymph node positivity and patients with cribriform pattern.

### Limitations

There are some limitations to this study. The first of these is the small number of patients in the study. Comparisons including the advanced disease subgroup among PCa patients would have been useful but were not performed. Naturally, response to chemotherapy and hormone therapy is not the subject of this study. Another limitation is the lack of comparison with other molecules that can show diagnosis, prognosis, and stage. According to the results, AUC is significant but remains in the ‘unsatisfactory’ range. Despite these limitations, its strongest feature is that it is the first study to investigate the relationship between PCa and sorcin.

The serum level of sorcin, which has been shown to be overexpressed in many cancer types, was found to be higher in PCa patients compared to the healthy male population. According to the results, high serum sorcin levels are observed in patients with high ISUP grade, positive lymph node, cribriform pattern, and BCR. Multicenter, long-term studies are needed to more clearly determine the place of sorcin in the diagnosis, treatment, and follow-up of PCa.

## Figures and Tables

**Table 1.** Comparison of Age, Serum PSA Levels, and Serum Sorcin Levels Between Groups

	Prostate cancer (n = 81)	Control (n = 67)	*P*
Age (years) Mean ± SD	63.65 ± 6.08	68.96 ± 6.25	**<.001**
PSA level (ng/mL) Mean ± SD	14.16 ± 19.85	1.47 ± 0.99	**<.001**
Sorcin level (ng/mL) Mean ± SD	1.72 ± 4.61	0.51 ± 0.26	**.02**

Independent sample *t*-test was used.

PSA, prostate-specific antigen.

**Table 2.** Comparison of Serum Sorcin Levels and Staining Percentages According to Clinical and Histopathological Findings of Prostate Cancer Patients

		Sorcin level (ng/mL)	*P*	Sorcin staining (%)	*P*
DRE finding	**−**	1.09 ± 1.84	.295	87.94 ± 28.27	.739
	**+**	2.18 ± 5.83		90.00 ± 25.79	
EPE	**−**	1.45 ± 2.12	.603	84.62 ± 31.53	.15
	**+**	1.98 ± 6.09		93.33 ± 20.91	
Surgical margin	**−**	1.45 ± 2.11	.43	88.93 ± 27.01	.917
	**+**	2.33 ± 7.74		89.60 ± 26.69	
Lymph node positivity	**−**	0.49 ± 0.16	**.018**	88.89 ± 26.94	.689
	**+**	1.88 ± 4.87		80.00 ± 40.00	
LVI	**−**	1.76 ± 4.80	.633	89.32 ± 26.24	.874
	**+**	1.35 ± 1.63		87.14 ± 34.02	
PNI	**−**	1.04 ± 1.57	.229	83.50 ± 34.83	.379
	**+**	1.95 ± 5.23		90.98 ± 23.57	
Cribriform pattern	**−**	1.13 ± 1.63	**.02**	84.52 ± 32.85	.268
	**+**	2.09 ± 5.72		92.00 ± 22.04	
Intraductal carcinoma	**−**	1.75 ± 4.84	.683	90.68 ± 24.22	.346
	**+**	1.48 ± 0.91		75.00 ± 43.43	
HGPIN	**−**	1.19 ± 1.99	.298	91.67 ± 24.19	.384
	**+**	2.30 ± 6.31		86.41 ± 29.33	
Biochemical recurrence	**−**	0.65 ± 0.66	**.049**	91.00 ± 24.68	.363
	**+**	2.09 ± 5.31		83.00 ± 30.93	

Independent sample t-test was used.

DRE, digital rectal examination, EPE, extraprostatic extension, HGPIN, high-grade prostatic intraepithelial neoplasia; LVI, lymphovascular invasion, PNI, perineural invasion.

**Table 3.** Comparison of Sorcin Levels and Staining Percentages According to ISUP Grades

	Sorcin Level		Sorcin Staining Percentage	
	Mean ± SD	*P*	Mean ± SD	*P*
TRUSG biopsy				
ISUP grade 1 (n = 32)	1.45 ± 1.79	**.013** **^a^**	84.69 ± 33.02	.702
ISUP grade 2 (n = 21)	1.31 ± 2.27		90.00 ± 22.58	
ISUP grade 3 (n = 12)	1.24 ± 2.63		93.33 ± 20.15	
ISUP grade 4 (n = 8)	0.62 ± 0.70		98.75 ± 3.54	
ISUP grade 5 (n = 8)	5.77 ± 13.46		88.75 ± 31.82	
Radical prostatectomy				
ISUP grade 1 (n = 13)	1.42 ± 1.98	.575	80.00 ± 39.16	.557
ISUP grade 2 (n = 24)	1.64 ± 2.68		86.67 ± 28.39	
ISUP grade 3 (n = 19)	0.94 ± 1.23		91.05 ± 23.78	
ISUP grade 4 (n = 8)	0.91 ± 0.32		97.50 ± 4.63	
ISUP grade 5 (n = 17)	3.34 ± 9.32		93.53 ± 22.06	

ANOVA test and Bonferroni post-hoc test were used.

ISUP, International Society of Urological Pathology; TRUSG, transrectal ultrasound guided.

^a^5 > 4 = 3 = 23 = 2 = 1 (post-hoc analysis).

## Data Availability

The data that support the findings of this study are available on request from the corresponding author.
